# Fundamental Properties and Self-Healing Performance of Repair Mortar with Solid Capsules Made Using Inorganic Reactive Powder

**DOI:** 10.3390/ma15051710

**Published:** 2022-02-24

**Authors:** Sung-Rok Oh, Kwang-Myong Lee, Sung Choi, Yun-Wang Choi

**Affiliations:** 1 R&D Team, Newjust Co., Ltd., Gwangmyeong 14348, Korea; 2 Department of Civil, Architectural and Environmental System Engineering, Sungkyunkwan University, Suwon 16419, Korea; leekm79@skku.edu; 3 Department of Civil Engineering, Kyungdong University, Yangju 26495, Korea; csomy1113@kduniv.ac.kr; 4 Department of Civil Engineering, Semyung University, Jecheon 27136, Korea; crete77@semyung.ac.kr

**Keywords:** repair mortar, healing performance, inorganic reactive powder, self-healing, solid capsules

## Abstract

Self-healing cement composites are generally produced by using materials such as inorganic powders, bacteria pellets, and microcapsules. Among them, inorganic powder-type healing materials tend to decrease in healing performance over time because they react relatively quickly. Accordingly, this study encapsulated self-healing inorganic reactive powders in solid capsules (SC) in order to delay their reaction. The capsule surface was coated with a membrane to prevent moisture from permeating it. SC were utilized to provide the self-healing effect to the repair mortar. SC were mixed at three rates (0%, 5%, and 10%) by the binder mass of the repair mortar. The fundamental properties, including rheology, table flow, strength, and length change, and the self-healing performance of the self-healing repair mortar mixes were investigated. It was found that the rheological and mechanical properties of the repair mortar decreased slightly as the amount of SC increased. On the other hand, for a crack width of 0.25 mm and crack inducing age of 28 days, the healing performance of repair mortar specimens containing SC was at least 20 pt% better than that of plain repair mortar after a healing period of 28 days.

## 1. Introduction

Recently, smart self-healing materials have emerged worldwide in the construction industry. Cracks occur in most concrete structures due to a variety of causes, such as shrinkage and mechanical loading, which decrease functionality, accelerate degradation, and reduce service life and sustainability of the structure [1]. Cracks in a concrete structure constructed with self-healing materials can be healed at an early stage of cracking, which can greatly reduce the time, effort, and cost required for general structural maintenance. In particular, self-healing technology has the potential to repair mechanical damage and cracks in concrete structures effectively, even in difficult-to-access structures [2,3,4]. For example, self-healing repair methods have been applied to an existing subway tunnel to stop water leaking from cracks [5].

Materials such as minerals [6], bacteria [7,8], superabsorbent polymers (SAPs) [9], and microcapsules [10,11] are being explored for use as self-healing materials. Inorganic materials improve the self-healing capacities of cement-based materials through the hydration of unhydrated cement [12], the generation of calcium carbonate via the carbonation reaction of Ca^2+^ [13], or the generation of C-S-H via pozzolanic reactions of fly ash or blast furnace slag [14]. The self-healing phenomenon of concrete using geomaterials occurs mainly due to swelling, expansion, and re-crystallization [6]. When a crack occurs, SAPs absorb the water that comes through the crack and expand to physically block the crack [9]. Bacteria heals cracks through a phenomenon in which CO_2_ generated by their metabolic activity forms CaCO_3_ crystals through reaction with Ca(OH)_2_ in a hardened cement paste [7,8].

Self-healing technology using capsules, which can contain a large amount of self-healing substances, has the advantage of selectively reacting to cracks. Self-healing capsules can be classified into solid capsules (SC) coated with a film, which are made by agglomeration of the powdery material [15,16], and microcapsules or macrocapsules encapsulated by a chemical method [11,17]. Accordingly, the capsule utilization technology can provide self-healing performance by employing a different reaction mechanism depending on the target material, and an appropriate core material phase can be selected [18]. In addition, many systems and techniques have been proposed to heal concrete cracks autonomically, such as modifying concrete by embedding microcapsules or hollow tubes with a suitable healing agent. When a crack occurs the shell of the capsule or the wall of the tube ruptures, the healing agent is released and reacts in the region of damage to produce new compounds, which seal the crack and/or bond the crack faces together [19].

The use of mineral admixtures as self-healing agents in cement-based composites has been studied extensively. However, if minerals are added directly to the cementitious matrix without any protection, they can immediately react, leading to a decrease in self-healing efficiency with additional side effects on the mechanical properties of cementitious composites. In order to overcome such a problem, several kinds of methods have been proposed. Choi et al. [15] fabricated crack self-healing solid capsules encapsulated with cement powder which can react with water in the event of breaking. Kishi et al. [20] investigated the crack healing capability of concrete incorporating granules of pozzolanic materials, Portland cement, and some specific admixtures and its feasibility in practice. Pan pelletization was used to produce pellets from three different powdered minerals as potential healing agents: reactive magnesium oxide (MgO), silica fume, and bentonite. Prototype pellets were then encapsulated in a polyvinyl alcohol (PVA)-based film coating [21,22]. The impregnation of lightweight aggregates by a liquid self-healing mineral and their subsequent encapsulation in a polymer-based coating layer was suggested as a method for improving the self-healing performance of concrete composites [10].

In this study, self-healing SC were manufactured by combining a core production process, in which a powdered expansive agent is granulated, with a coating process to protect the surfaces of the granulated particles. Hydration of the core materials can be delayed by maintaining the moisture barrier performance of the wall material until SC are broken by cracking of the concrete, so that it is possible to retain healing performance even after significant aging. SC mixed with repair mortars might be destroyed during re-cracking after repairing, causing a hydration reaction with the surrounding moisture, and can induce a crack healing reaction through the hydration product. Therefore, the utilization of optimal dosages of SC could have great potential for applications in repairing cracked concrete under the water leakage of water reservoirs, underground structures, and tunnels.

The quality and self-healing performance of repair mortar using SC were evaluated through the experiment. Concrete mixed with SC made by using expansive agents as core materials was tested to evaluate its fundamental properties such as rheological properties, strength, and length change. In addition, cracks were induced in self-healing repair mortar specimens at 28 days and 91 days, and the healing performance of the specimens was evaluated throughout the 28-day healing period. Through the experimental results, the optimal mixing ratio of SC for self-healing repair mortar was suggested.

## 2. Materials and Test Methods

### 2.1. Solid Capsules

The healing material used in the SC is an inorganic reactive powder mixture that reacts by hydration. The inorganic reactive powder used for producing SC consisted of mixing a hauyne-based expansion material, which was calcium sulfoaluminate (CSA; made by Denka Co., Ltd., Tokyo, Japan), and general anhydrite (CaSO_4_). 

Equation (1) shows the reaction mechanism of the healing materials. CSA generates ettringite or calcium hydroxide by hydration reaction, which induces expansion, and CaSO_4_ promotes crystal growth and generates hexagonal plate crystals. Therefore, the main healing products are composed of ettringite (C-S-H) and calcium hydroxide (Ca(OH)_2_).

**Figure 1:**
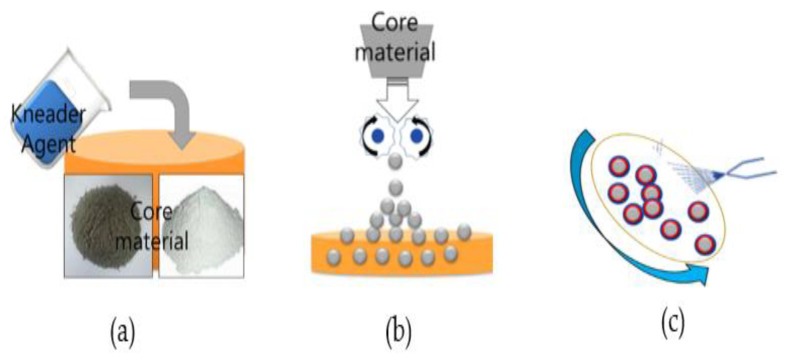
Manufacturing process of SC: (**a**) mixing of kneader agent, (**b**) granulation, and (**c**) coating of wall materials.

The manufacturing process of the SC consists of three steps: mixing healing materials (Figure 1a), granulating the healing materials (Figure 1b), and coating and drying SC (Figure 1c). The mixing ratio of CSA to CaSO_4_ was 70:30, following the previous study [16], and SC ranging from 0.85 to 1.2 mm were used to produce the self-healing repair mortar [15,16]. The surface of SC was coated by spraying polyurethane to delay the reaction of the core material.

(1) $$\mathrm{CSA}\left(3{\mathrm{CaO}}_{2}\times 3{\mathrm{Al}}_{2}O_{3}\times \mathrm{CaSO}\right)+\mathrm{anhydrous}\;\mathrm{gypsum}\left({\mathrm{CaSO}}_{4}\right)\times \mathrm{Free}\;\mathrm{CaO}+{\mathrm{xH}}_{2}O\;\to \mathrm{Ettringite}\left(C-A-\;\overset{-}{S}-H+\mathrm{Ca}{\left(\mathrm{OH}\right)}_{2}\right)$$

### 2.2. Repair Mortar

The mixing proportions of the repair mortar with a target compressive strength of 40 MPa were W:B:S = 0.4:1.0:1.5, as shown in Table 1. The binder consisted of ordinary Portland cement (C), metakaolin (M), and zeolite (Z), and the fine aggregate was prepared by blending three sizes of silica sand with a density of 2.60 g/cm^3^. The three sizes of silica sand are 1.5–2.4 mm (#3), 0.7–1.2 mm (#5), and 0.35–0.7 mm (#6). In addition, short polymer fibers (polyvinyl alcohol, PVA) with 0.1% of the total volume of the repair mortar were used to improve tensile strength and to reduce drying shrinkage of the mortar. The PVA fibers used were approximately 6–8 mm in length and consisted of single yarn fibers with a tensile strength greater than 450 MPa. SC accounted for 0%, 5%, and 10% by mass of the binder in Plain, SC5, and SC10 mixes, respectively.

### 2.3. Test Methods for Fundamental Properties of Repair Mortar

#### 2.3.1. Rheological Properties

The rheology of the repair mortar was evaluated by referring to the results of previous studies because there is no related regulation [23,24]. The rheology measurement was carried out using a Brookfield DV-III Ultra mortar viscometer with a modified chamber size.

**Figure 2:**
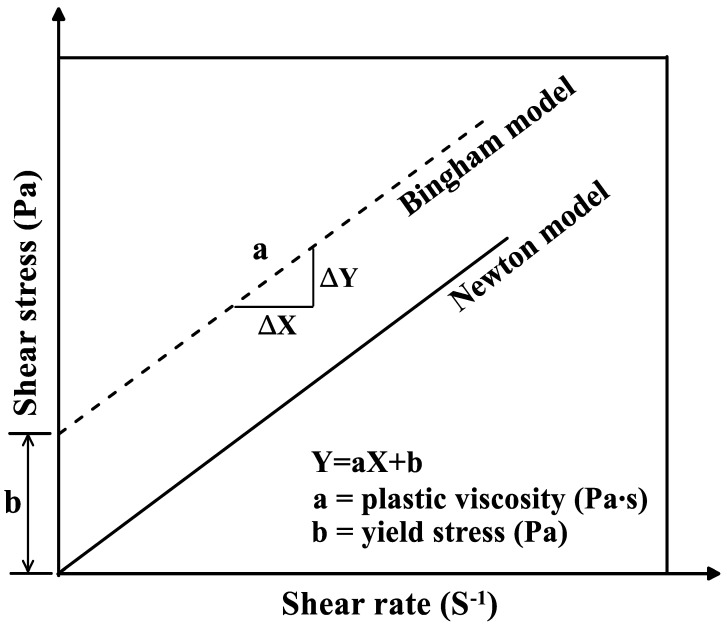
Rheological analysis method.

In general, the fluidity of mortar is determined through a flow test, but the flow test results have difficulty expressing the rheological properties of the semi-plastic mortar. The rheological properties are related to the workability and viscosity of the mortar. In general, the viscosity of fluid, such as water and oil, is characterized using a Newton model, as shown in Figure 2. However, as mortar is not in a pure liquid phase, it is interpreted as a Bingham model that does not flow until a certain external force is applied. The Bingham model can be obtained by the correlation between shear rate and shear stress measured by the viscometer. The rheological properties of repair mortars were evaluated through the plastic viscosity and the yield stress which are the slope and the y-intercept of the Bingham model, respectively [23,24].

The flow and air content of the repair mortar were measured according to ASTM C1437 [25] and ASTM C185 [26], respectively.

#### 2.3.2. Mechanical Properties

The compressive strength of the repair mortar was measured at 3, 7, and 28 days of age according to ASTM C109 [27]. For the compressive test, 50 mm cube specimens were prepared and cured at a temperature of 20 °C and a relative humidity of 60% for one day. After demolding, all specimens were cured in water with a temperature of 20 °C. The bond strength of the repair mortar was measured at 28 days according to ASTM C1583 [28]. The flexural strength at 28 days and length change of the repair mortar were also measured according to ASTM C348 [29] and ASTM C157 [30], respectively. Three kinds of strength were measured on a total of six specimens, respectively, and four measured values excluding the maximum and minimum values were averaged and used as the measurement value. The length change was the average value of the measurements of three specimens.

### 2.4. Test Methods for Healing Performance of Repair Mortar

#### 2.4.1. Water Permeability Test

**Figure 3:**
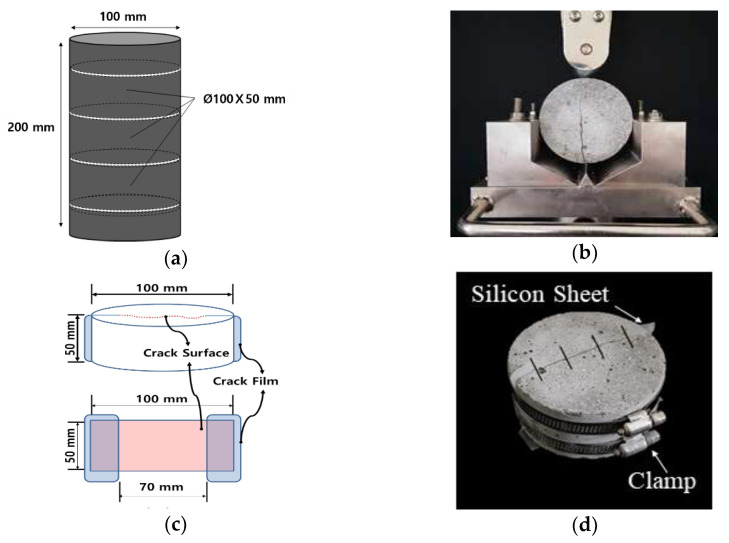
Preparation of cracked specimens: (**a**) slicing into three disc specimens, (**b**) split specimen, (**c**) crack induction, and (**d**) specimen bound together with two steel bands.

A constant water head permeability test was adopted to measure the water flow rate of the crack-induced specimen and to evaluate the self-healing performance of repair mortars [31]. For the water permeability test, cracked specimens were produced through several steps. First, mortar cylinders (Ø100 × 200 mm) were prepared, as shown in Figure 3a. Next, these cylinders were demolded after 24 h and were cured in a water bath at 20 °C until they reached the crack-inducing age.

Once the cracking age was attained, a cylinder was sliced into three disc specimens (Ø100 × 50 mm) and then split into two semi-circular sections, as shown in Figure 3b. Then, a flexible silicone rubber sheet with varying thickness was attached to both ends of the cracked sections to induce a crack of specified width, as shown in Figure 3c. The actual lengths of the cracks were approximately 70 mm. Finally, the split specimens were bound together using stainless steel bands to maintain the desired crack widths, as shown in Figure 3d. For each specimen, cracks were induced at 28 and 91 days, with crack widths ranging from 0.2–0.25 mm. After the specimens were prepared, the widths and lengths of cracks were measured using a microscope (EGVM-35M, EG Tech, Anyang, Korea).

**Figure 4:**
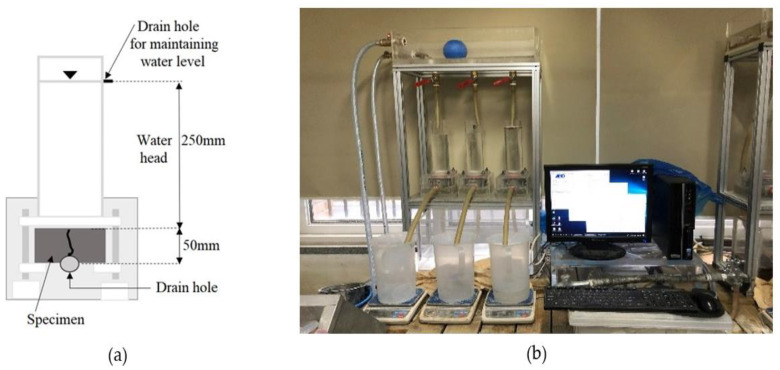
Water permeability test apparatus: (**a**) schematic diagram and (**b**) test setup.

The cracked specimens were cured in a water bath at 20 °C during the healing period. As there is no specific standard for the water temperature during the healing period, a water curing temperature of 20 °C was adopted [27,31]. The water permeability test was conducted after healing periods of 0, 7, 14, 21, and 28 days. Figure 4a shows the test schematic diagram of the water permeability test. Figure 4b shows the water permeability test apparatus for the cracked disc specimens. The amount of water coming out of the test equipment was measured for 7 min after the water head and water flow stabilized. The water flow rate in units of mL/(mm·min) was obtained by dividing this amount of discharged water by the test duration (min) and crack length (mm).

Through the water permeability test, the healing index, *SH_q_*, can be calculated using the water flow rate reduction ratio as follows [2,31,32,33]:

(2) $$SH_{q}=\left[1-\frac{q\left(t\right)}{q0}\right]\times 100\left(\%\right),$$

where *q*0 is the initial water flow rate measured just after the specimen is cracked, without any healing effect, and *q*(*t*) is the water flow rate after a healing period, *t*.

#### 2.4.2. Crack Monitoring and Analysis of Healing Product

**Figure 5:**
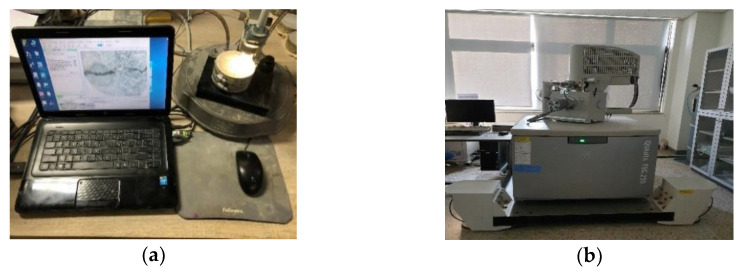
Healing product observation methods: (**a**) microscope and (**b**) SEM.

Crack monitoring was performed at 100× magnification using a microscope (EGVM-35B, EG Tech, Korea). The crack closing level of the crack surface by the reaction product was monitored according to the healing period. Healing product samples were collected for healing product analysis, which was performed using a scanning electron microscope (SEM, Quanta 250 FEG, FEI co., Hillsboro, OR, USA). Figure 5 shows the optical microscope and SEM used for the observation of healing products.

## 3. Results and Discussion

### 3.1. Fundamental Properties of Repair Mortar

#### 3.1.1. Rheological Properties

**Figure 6:**
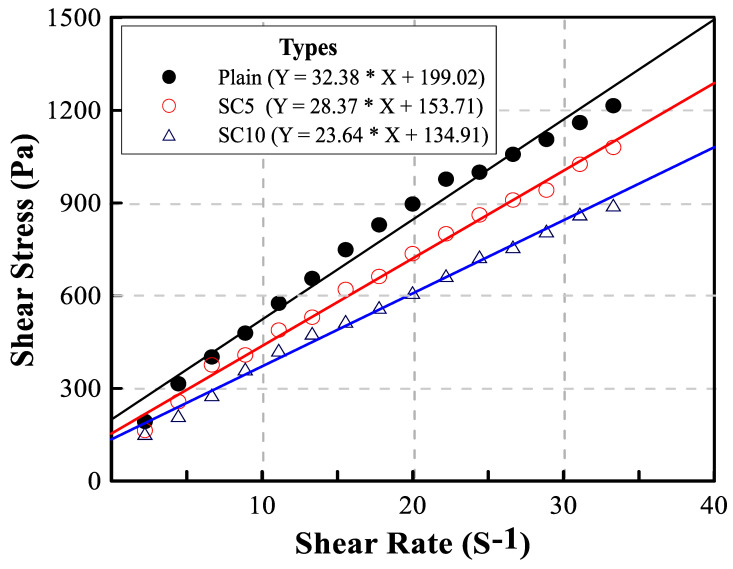
Rheology curve of three types of repair mortar.

Figure 6 shows the rheology test results analyzed with the Bingham model. The slope of the rheology curve obtained by linear regression analysis tended to decrease as the amount of SC increased. The slope of the rheology curve represents the plastic viscosity, and the y-intercept value represents the yield stress [23,24].

Table 2 summarizes the plastic viscosity and yield stress of the three types of repair mortar. Both the plastic viscosity and yield stress of SC5 and SC10 decreased considerably as the amount of SC increased. The plastic viscosity and yield stress of SC5 were 12% and 23% lower than that of Plain, respectively. Table 2 also summarizes the flow and air content of the repair mortars. The flow of SC5 and SC10 showed a tendency to decrease slightly compared to Plain, and the flow losses of SC5 and SC10 at 60 min were 8 mm and 20 mm, respectively. The air contents of all mixes were ~7%, which were not influenced by SC [15].

#### 3.1.2. Mechanical Properties

Table 3 summarizes the test results of the mechanical properties of the repair mortars. The compressive strength, bond strength, and flexural strength of the repair mortars decreased as the amount of SC increased due to the weak SC. As a typical result, the 28-day compressive strengths of SC5 and SC10 were reduced by 5.2% and 10.6% compared to Plain, respectively. Although the target strength of 40 MPa was achieved, the amount of SC should be determined in consideration of the strength reduction as well as the self-healing effect of the repair mortar with SC for practical use. The bond strength and flexural strength showed similar trends to the compressive strength. The length changes of SC5 and SC10 were 11.4% and 22.8% lower than that of Plain, respectively, due to the expansion effect of SC [15].

### 3.2. Healing Performance of Repair Mortar

#### 3.2.1. Water Flow Rate

**Figure 7:**
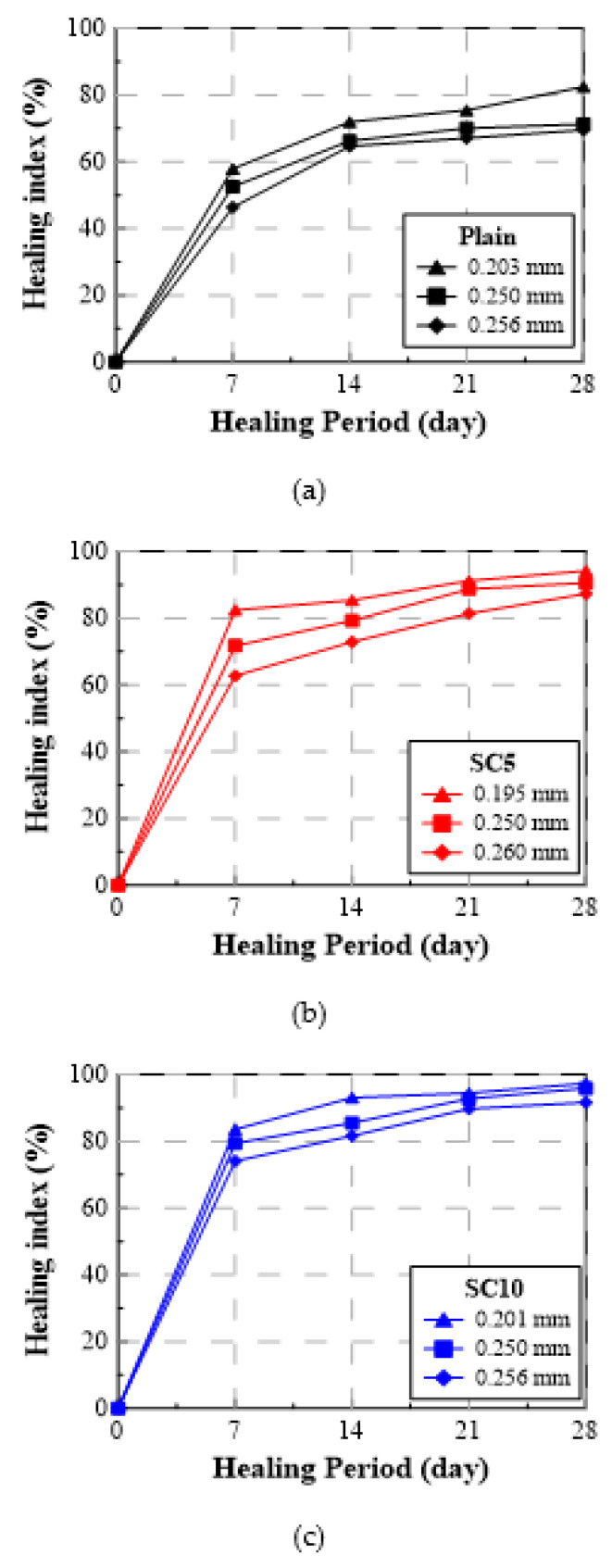
Healing index (%) vs. healing period (crack induction age: 28 days) for three types of repair mortar: (**a**) Plain, (**b**) SC5, and (**c**) SC10.

**Figure 8:**
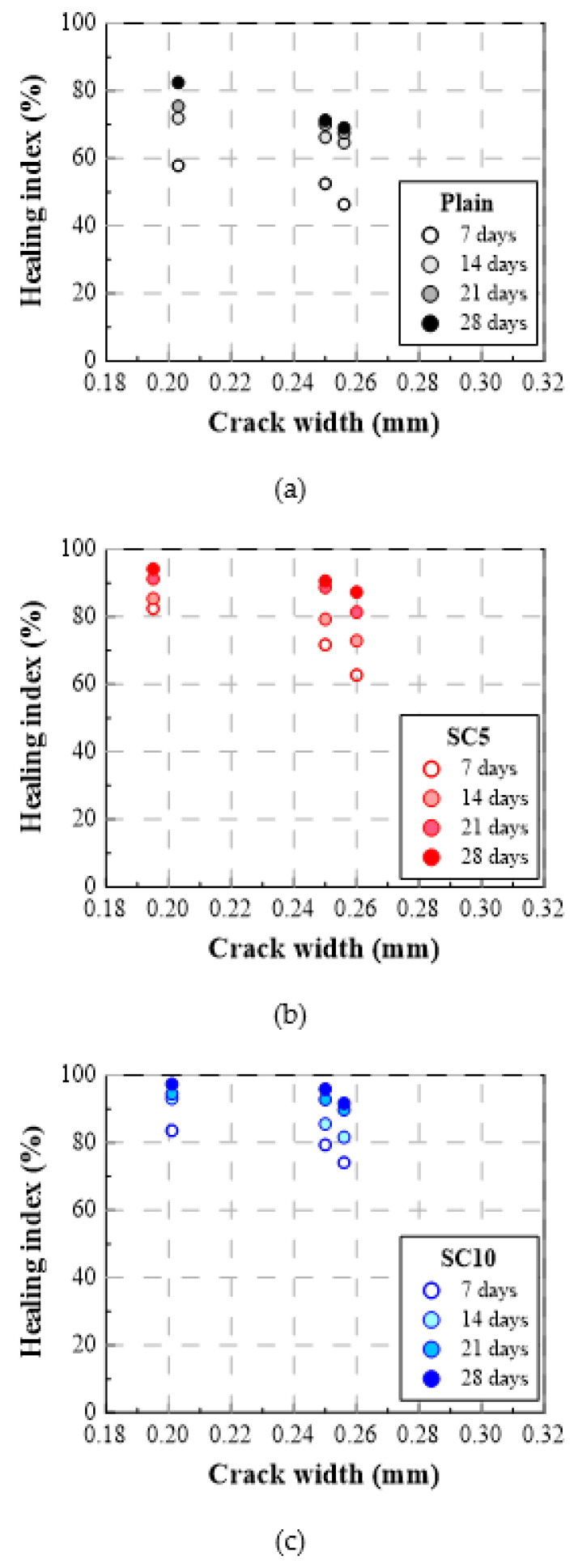
Healing index (%) vs. crack width (crack induction age: 28 days) for three types of repair mortar: (**a**) Plain, (**b**) SC5, and (**c**) SC10.

Table 4 summarizes the water flow rate and healing index of Plain, SC5, and SC10 according to the healing period. The water flow rate gradually decreased as the healing period elapsed, the unit runoff decreased rapidly at the beginning, and the water flow rates of SC5 and SC10 decreased more significantly compared to Plain. Figure 7 and Figure 8 show the test results for the crack inducing age of 28 days as the healing index (%) vs. healing period and healing index (%) vs. crack width, respectively. As shown, the healing index increased rapidly during the initial seven days of the healing period, and then increased more slowly during days 7 through 28 [31].

#### 3.2.2. Healing Index

When the crack inducing age was 28 days and the healing period was 7 days, the healing indices of SC5 and SC10 with the crack width of 0.25 mm were 72% and 79%, respectively, improved by 19 pt% and 26 pt% compared to Plain’s healing index of 53%. The healing index of SC10 increased by 7 pt% compared to that of SC5, confirming that SC had a significant healing effect. Under the same crack inducing age, the healing indices of SC5 and SC10 with the healing period of 28 days were 91% and 96%, respectively, representing improvements of 20 pt% and 25 pt% compared to Plain’s healing index of 71%. The healing index of SC10 increased by only 5 pt% compared to SC5, so the difference in the healing performance between SC5 and SC10 was not much when a crack was induced at 28 days. When the crack width increased from 0.20 mm to 0.25 mm and the healing period was 28 days, the healing indices of SC5 and SC10 decreased only 3 pt% and 1 pt%, respectively, while the healing index of Plain decreased 11 pt%. This means that even if the crack width increases, the healing effect of SC could be maintained.

**Figure 9:**
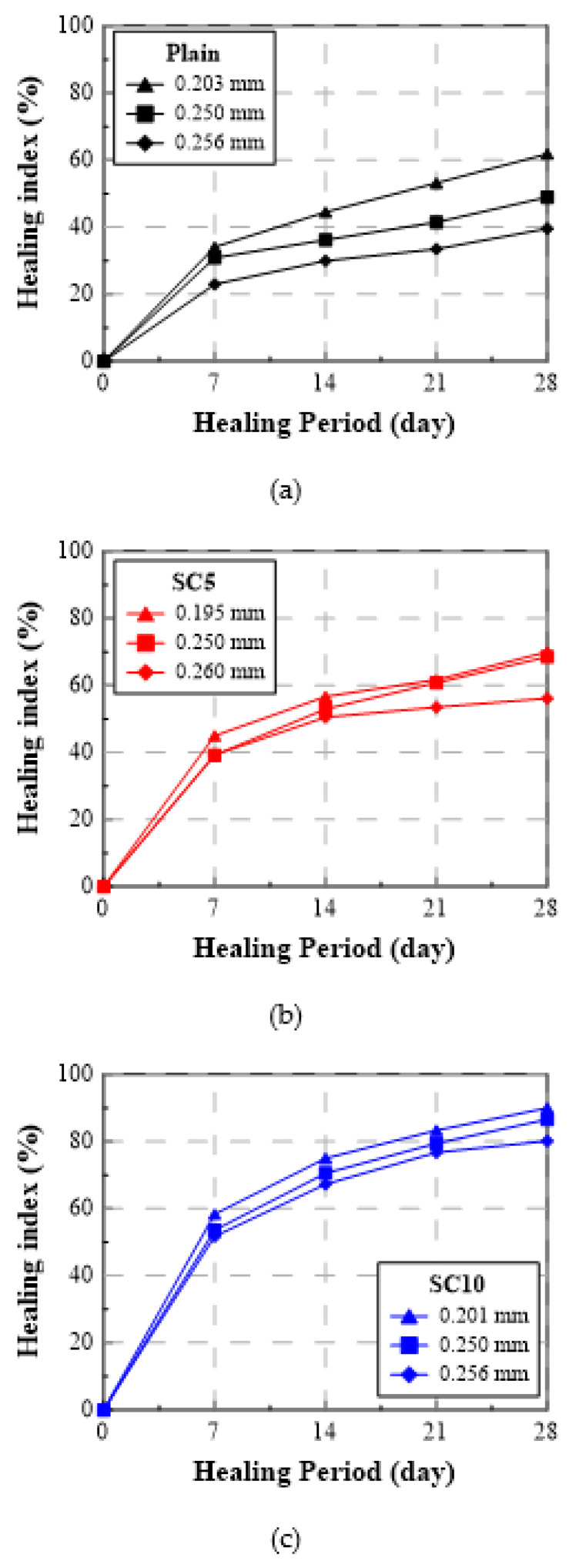
Healing index (%) vs. healing period (crack induction age: 91 days) for three types of repair mortar: (**a**) Plain, (**b**) SC5, and (**c**) SC10.

**Figure 10:**
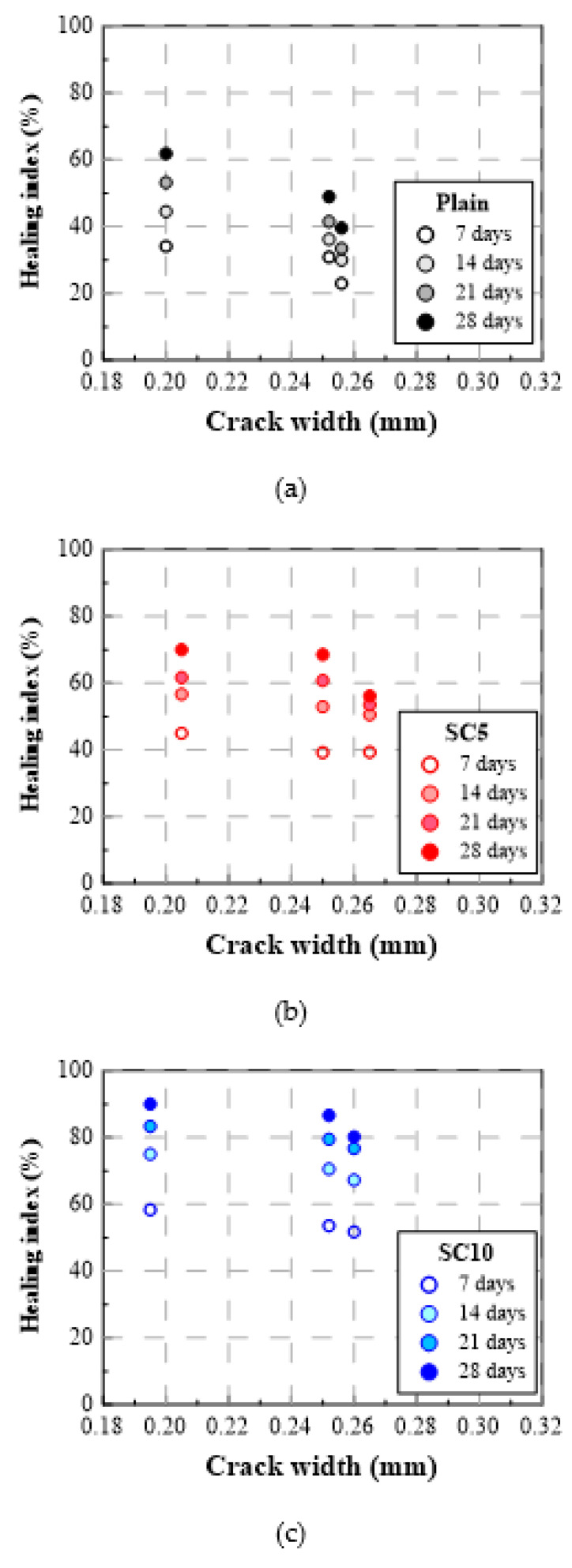
Healing index (%) vs. crack width (crack induction age: 91 days) for three types of repair mortar: (**a**) Plain, (**b**) SC5, and (**c**) SC10.

Figure 9 and Figure 10 show the test results of inducing cracks at 91 days as the healing index (%) vs. healing period and healing index (%) vs. crack width, respectively. As shown in Table 4 and Figure 9 and Figure 10, the healing indices for a crack inducing age of 91 days were somewhat different from those for a crack inducing age of 28 days. First, the healing indices of Plain, SC5, and SC10 for a crack inducing age of 91 days decreased by at least 20 pt% compared to the crack inducing age of 28 days regardless of the initial crack width. Second, SC10, which contained the most SC, exhibited the least reduction in the healing performance after a healing period of 7 days, and the healing index of SC10 was rapidly improved over the healing period.

When the crack induction age was 91 days and the crack width was 0.25 mm, the healing index of Plain after a healing period of 7 days was only 31%, and those of SC5 and SC 10 were 39% and 54%, respectively. Accordingly, the healing index of SC5 and SC10 increased by 8 pt% and 23 pt% compared to Plain [15,16,31]. Moreover, after a healing period of 28 days, the indices of SC5 and SC10 increased by 20 pt% and 37 pt% compared to Plain. These implied that the amount of SC became more effective on the self-healing performance as the crack inducing age increased.

**Figure 11:**
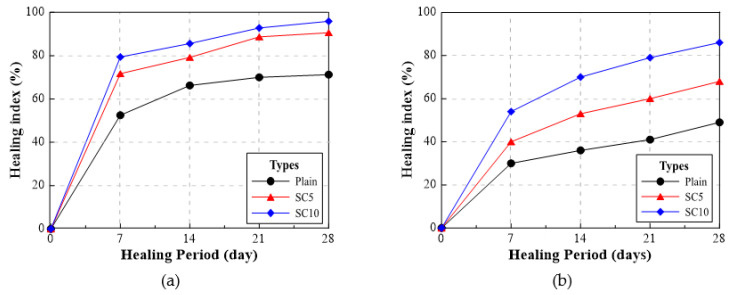
Healing index vs. healing period of three types of repair mortar with a crack width of 0.25 mm for crack induction ages of (**a**) 28 days and (**b**) 91 days.

Figure 11a,b shows the correlation between the healing period and the healing index of the three types of repair mortar specimens with the crack width of 0.25 mm for crack induction ages of 28 days and 91 days, respectively. As shown in Figure 11a, when a crack was induced at 28 days, the healing index increased rapidly during the first seven days of the healing period, while when a crack was induced at 91 days, the healing index increased gradually, as the specimens cracked at 91 days had relatively less unhydrated binders than the specimens cracked at 28 days [31].

As shown in Figure 11b, when a crack was induced at 91 days of age, the healing index of Plain was significantly lower than that of SC10. It is found from such a result that the hydration of the core expansive materials was delayed by maintaining the moisture barrier performance of the capsule membrane until crack induction, so that the healing performance of self-healing repair mortar can be retained longer [15].

However, if a large amount of SC is added to the repair mortar as a self-healing material, the quality of the repair mortar tends to be changed considerably, so it is necessary to determine the optimal amount of SC in order to satisfy not only the required quality and the target healable crack width but also the economic efficiency.

#### 3.2.3. Crack Monitoring

**Figure 12:**
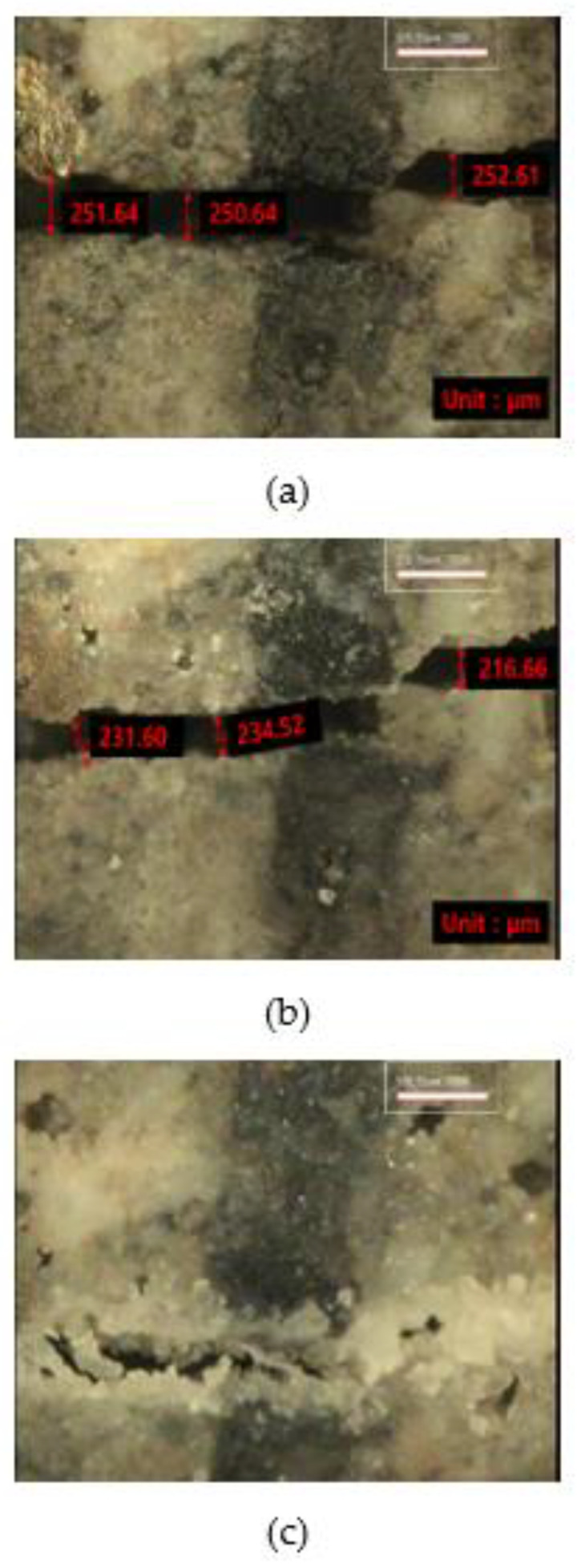
Changes in a surface crack of Plain specimen with healing period: (**a**) 0 days, (**b**) 7 days, and (**c**) 28 days.

**Figure 13:**
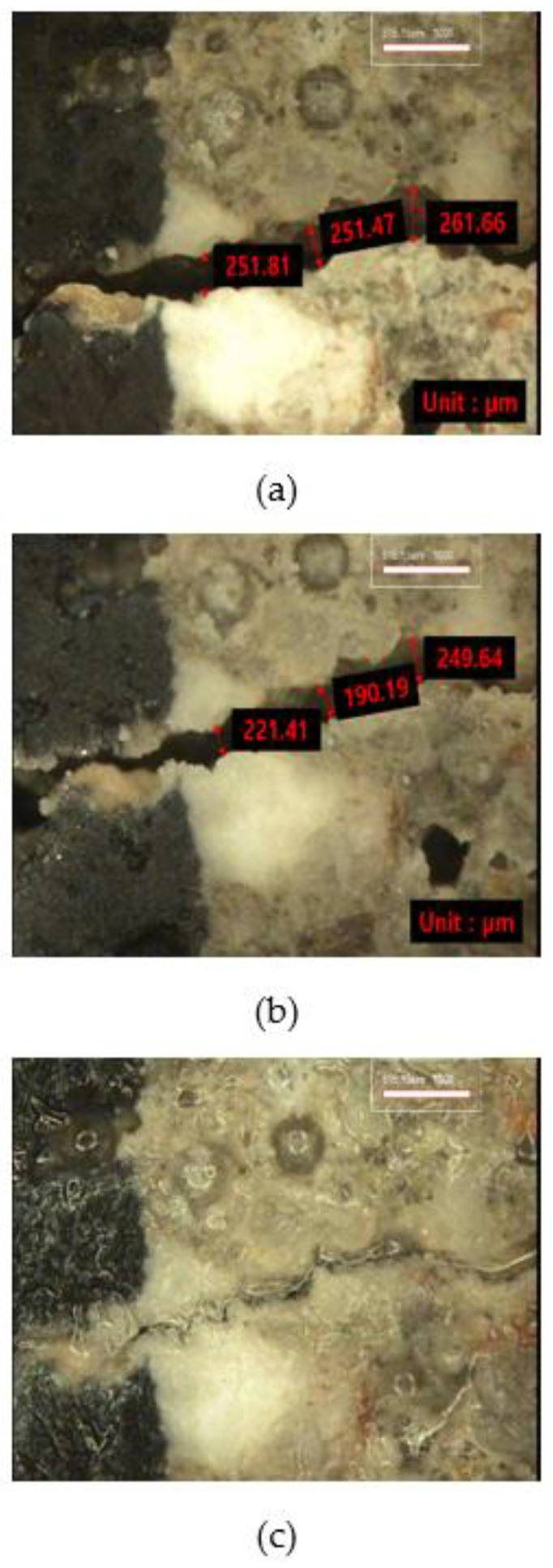
Changes in a surface crack of SC5 specimen with healing period; (**a**) 0 days, (**b**) 7 days, and (**c**) 28 days.

**Figure 14:**
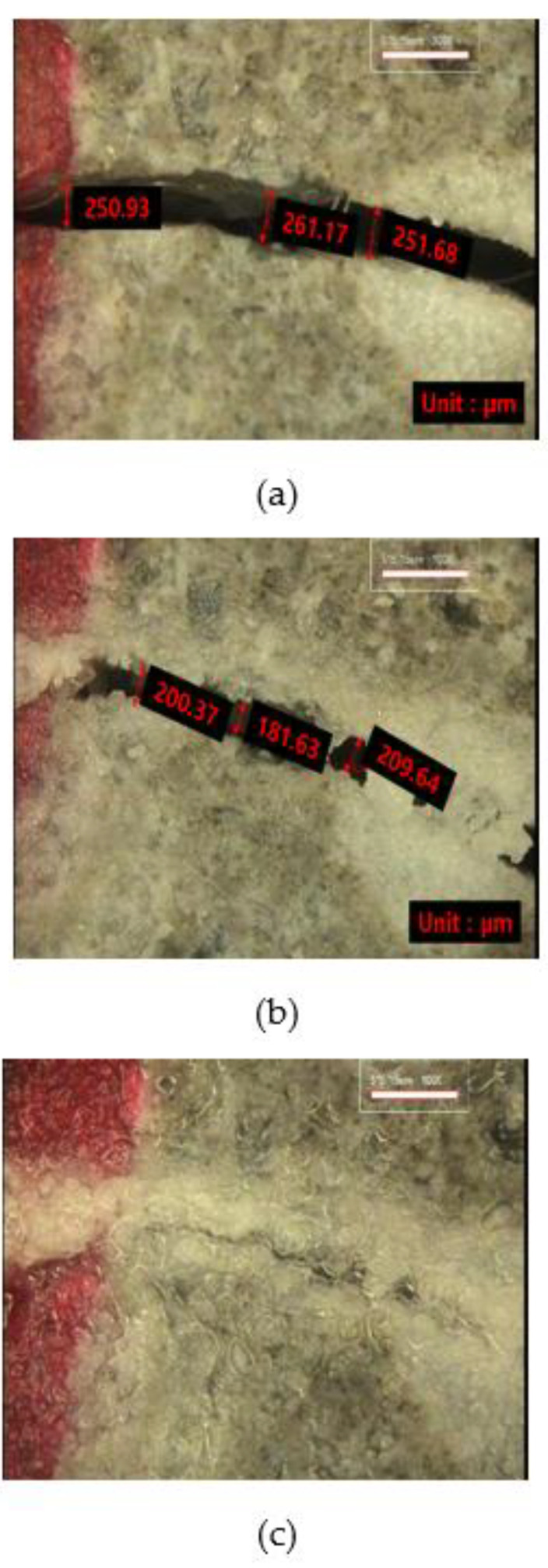
Changes in a surface crack of SC10 specimen with healing period: (**a**) 0 days, (**b**) 7 days, and (**c**) 28 days.

**Figure 15:**
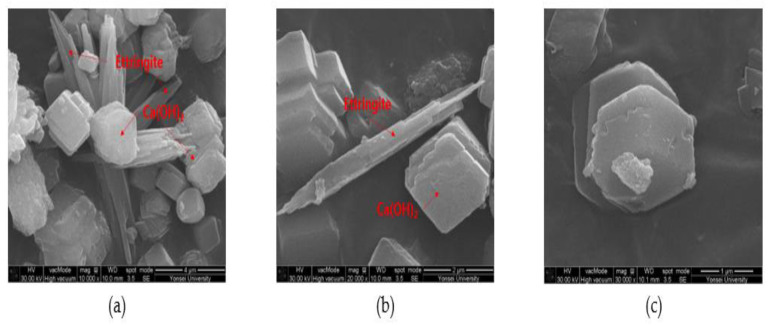
Healing products of SC by SEM: (**a**) 10,000×, (**b**) 20,000×, and (**c**) 30,000×.

Figure 12, Figure 13 and Figure 14 show the monitoring results of the crack surface for the three types of repair mortar specimens. As a result of monitoring the crack surface of the specimen with a crack width of 0.25 mm, it was confirmed that most of the cracks were healed by the healing products appearing on the surface after the specific healing period. Figure 15 shows the scanning electron microscopy (SEM) images of the healing products. It was observed that the healing products consisted of hexagonal crystals of calcium hydroxide and needle-like crystals of C-S-H [34,35].

## 4. Conclusions

This study investigated the rheological and mechanical properties and self-healing performance of repair mortars containing SC made using an inorganic reactive powder. The conclusions are as follows:

1. In terms of the rheological properties of the repair mortars containing SC, the plastic viscosity and yield stress decreased with the addition of SC, and the flow tended to decrease as well. The strength of the repair mortar tended to decrease with the addition of SC. The effect of SC on the air content was not significant.
2. Through the water permeability test on repair mortars containing SC, it was found that the healing performance increased rapidly during the initial seven days of the healing period and increased more gradually thereafter. The healing performance of repair mortars containing SC increased as the amount of SC increased, indicating that the addition of SC could significantly increase the healing performance.
3. When cracks were induced after 91 days of age, the healing performance of repair mortars was improved by adding SC. Accordingly, it was confirmed that SC can preserve healing performance even after a long time because they are coated with a membrane material to delay the hydration of core materials.
4. Consequently, it is necessary to determine the optimal SC amount for self-healing repair mortar by considering not only the healing parameters, such as the target crack width, healing period, and healing index, but also the quality of the repair mortar, including rheological and mechanical properties. Through these results, the optimal mixing ratio of SC considering the fundamental properties and healing performance of the repair mortar might be 5% by mass of binder.

## Figures and Tables

**Table 1:** Mix proportions of the repair mortar.

Mix Type	Water	Binder			Fine Aggregate			PVA (Volume %)	SC
		1.0			1.5				
		C	M	Z	#3	#5	#6		
Plain	0.4	0.88	0.06	0.06	0.40	0.70	0.40	0.1	-
SC5	0.4	0.88	0.06	0.06	0.40	0.70	0.40	0.1	0.05
SC10	0.4	0.88	0.06	0.06	0.40	0.70	0.40	0.1	0.10

**Table 2:** Test results of rheological properties and air content of three types of repair mortar.

Mix Type	Plastic Viscosity (Pa·s)	Yield Stress (Pa)	Flow (mm)			Air Content (%)
			0 min	30 min	60 min	
Plain	32.38	199.02	200	195	190	7.06
SC5	28.37	153.71	195	190	187	7.01
SC10	23.64	134.91	190	187	170	6.90

**Table 3:** Test results of strengths and length change of three types of repair mortar.

Mix Type	Compressive Strength (MPa)			Bond Strength (MPa)	Flexural Strength (MPa)	Length Change (×10^−6^ με)
	3 Days	7 Days	28 Days	28 Days	28 Days	28 Days
Plain	24.3	38.1	52.1	2.0	10.5	−350
SC5	22.2	36.0	49.4	1.8	10.3	−310
SC10	20.7	33.1	46.6	1.5	10.0	−270

**Table 4:** Water flow rate and healing index.

Type	Crack Induction (Days)	Crack Width (mm)	Water Flow Rate (mL/(min × mm))					Healing Index, SH_q_ (Equation (2))			
			0d	7d	14d	21d	28d	7d	14d	21d	28d
Plain	28	0.203	0.57	0.24	0.16	0.14	0.10	0.58	0.72	0.75	0.82
		0.250	0.80	0.38	0.27	0.24	0.23	0.53	0.66	0.70	0.71
		0.256	0.82	0.44	0.29	0.27	0.24	0.46	0.65	0.67	0.68
	91	0.200	0.58	0.38	0.32	0.27	0.22	0.34	0.44	0.53	0.62
		0.252	0.94	0.65	0.55	0.47	0.39	0.31	0.36	0.41	0.49
		0.265	1.14	0.88	0.80	0.76	0.69	0.23	0.30	0.33	0.40
SC5	28	0.195	0.68	0.12	0.10	0.06	0.04	0.82	0.85	0.91	0.94
		0.250	1.06	0.30	0.22	0.12	0.10	0.72	0.79	0.89	0.91
		0.260	1.18	0.44	0.32	0.22	0.15	0.63	0.73	0.81	0.87
	91	0.205	0.60	0.33	0.26	0.23	0.18	0.45	0.57	0.62	0.70
		0.250	1.02	0.62	0.48	0.40	0.32	0.39	0.53	0.61	0.69
		0.265	1.14	0.69	0.56	0.53	0.50	0.39	0.50	0.54	0.56
SC10	28	0.201	0.80	0.13	0.06	0.04	0.02	0.84	0.93	0.94	0.97
		0.250	0.97	0.20	0.14	0.07	0.04	0.79	0.85	0.93	0.96
		0.256	1.20	0.24	0.22	0.12	0.10	0.74	0.82	0.89	0.92
	91	0.195	0.60	0.25	0.15	0.10	0.06	0.58	0.75	0.83	0.90
		0.252	1.12	0.52	0.33	0.23	0.15	0.54	0.71	0.79	0.86
		0.260	1.16	0.56	0.38	0.27	0.23	0.52	0.67	0.76	0.80

## Data Availability

Data available on request due to restrictions, e.g., privacy or ethical.
